# Effects of Brown Seed Coat Retention and Thermal Processing on Nutritional Composition, Bioactive Compounds, Antioxidant Activity, and Functional Properties of Jackfruit Seed Flour

**DOI:** 10.3390/foods15142529

**Published:** 2026-07-17

**Authors:** Theeraphan Chumroenphat, Nonthiwat Taesuk, Nidthaya Seephua, Apichaya Bunyatratchata

**Affiliations:** 1 Cosmetic Science and Spa Program, Faculty of Thai Traditional and Alternative Medicine, Ubon Ratchathani Rajabhat University, Mueang Ubonratchathani, Ubonratchathani 34000, Thailand; theeraphan.c@ubru.ac.th; 2 Department of Biology, Faculty of Science, Mahasarakham University, Kantarawichai, Maha Sarakham 44150, Thailand; nonthiwat.t@msu.ac.th; 3 Sustainable Approaches for Materials, Agriculture, and Health Technology (SAMAHT) Research Unit, Mahasarakham University, Kantarawichai, Maha Sarakham 44150, Thailand; 4 Department of Food Technology and Nutrition, Faculty of Technology, Mahasarakham University, Kantarawichai, Maha Sarakham 44150, Thailand; nidthaya.s@msu.ac.th; 5 Research Unit of Thai Food Innovation (TFI), Mahasarakham University, Kantarawichai, Maha Sarakham 44150, Thailand

**Keywords:** jackfruit seed flour, brown seed coat, thermal processing, phenolic compounds, flavonoids, antioxidant activity, β-glucan, functional properties

## Abstract

Jackfruit seed is an underutilized by-product with potential as a functional ingredient. This study investigated how brown seed coat retention and thermal processing affect the color, nutritional composition, β-glucan content, bioactive profiles, antioxidant activity, and functional properties of jackfruit seed flour. Seeds were processed as raw, boiled, or steamed samples, with or without the brown seed coat retained, using rice and wheat flours as references. Brown seed coat retention reduced lightness and increased redness. Jackfruit seed flour generally showed higher ash, protein, and fiber contents than reference flours, while seed coat retention further enhanced fiber content. β-glucan content ranged from 56.11 to 286.71 mg/g. Brown seed coat samples showed the highest TPC, TFC, and antioxidant activity, followed by brown seed coat-retained flour samples. Thermal processing reduced bioactive compounds and antioxidant activity; however, steaming better retained bioactive compounds, antioxidant activity, and β-glucan content in brown seed coat-retained flour than boiling. Heatmap analysis revealed cinnamic acid, gentisic acid, myricetin, and quercetin as major compounds. Functional analysis showed higher WSI and WHC than reference flours, while OHC and WSC were generally comparable. These findings suggest the potential of brown seed coat-retained jackfruit seed flour for further development as a functional ingredient.

## 1. Introduction

Jackfruit (*Artocarpus heterophyllus* Lam.) is a tropical fruit widely cultivated across Asia, Africa, and South America. In Thailand, it is extensively grown, covering approximately 9094 hectares across 61 provinces, with a total production of about 95,031 tons reported in 2024 [1]. The fruit is recognized for its distinctive flavor, high nutritional value, and substantial production volume. Jackfruit comprises several components, including the edible bulbs, peel, fibrous pulp, and seeds [1]. In addition to the edible pulp, jackfruit processing generates a considerable quantity of by-products, particularly seeds, which represent approximately 15–20% of the total fruit weight. Despite their abundance, these seeds are often discarded as waste, even though they are rich in starch, protein, minerals, and various bioactive compounds [2,3]. Currently, the incorporation of fruit by-products into food formulations has gained increasing attention as a strategy to improve nutritional value, reduce waste, and promote the development of value-added food products [4,5]. However, the use of jackfruit by-products, especially seeds, remains limited, presenting opportunities for their further development in food applications.

Recent research has increasingly focused on the recovery and utilization of fruit seeds as sources of functional ingredients. Jackfruit seed flour has been incorporated into various food products, particularly bakery items such as bread, cookies, cakes, biscuits, and doughnuts, as well as extruded products [6,7,8,9,10]. These applications demonstrate the feasibility of using jackfruit seed flour as an alternative ingredient in food formulations. Moreover, jackfruit seeds have been reported to contain bioactive compounds, such as phenolic compounds and flavonoids, which exhibit antioxidant properties associated with potential health benefits [6]. In addition, the seed kernel has been shown to possess higher antioxidant activity than the fruit pulp [11]. Collectively, these findings highlight the potential of jackfruit seed flour as a promising functional ingredient in food products.

Structurally, jackfruit seeds consist of fleshy white cotyledons enclosed by a thin brown seed coat, known as the spermoderm, and surrounded by a white aril [12]. In most conventional processing methods for producing jackfruit seed flour or starch, the brown seed coat (spermoderm) is typically removed prior to further processing [7,13,14]. However, the seed coat may contain bioactive compounds, including phenolic compounds and flavonoids, which could contribute to antioxidant activity. Natural antioxidants play an important role in reducing oxidative stress, which is closely associated with the development of chronic diseases such as diabetes, cardiovascular disorders, and cancer [15]. Nevertheless, limited information is available regarding the functional characteristics, bioactive compound profiles, and antioxidant properties of jackfruit seed flour produced with the brown seed coat retained. Therefore, further investigation is needed to evaluate the potential benefits of brown seed coat retention during flour production and to better understand its contribution to the nutritional and functional properties of jackfruit seed flour.

Processing methods applied to seeds, such as boiling or steaming, can also strongly influence their bioactive compounds and functional characteristics. Different processing conditions may result in variations in phenolic and flavonoid profiles at the level of individual compounds [16]. Thermal processing has also been reported to affect total phenolic content (TPC), total flavonoid content (TFC), and antioxidant activity [17,18]. Phenolic compounds are thermally unstable and may be lost in substantial amounts during heating [19]. Furthermore, heat treatment may cause structural changes in phenolic compounds and the loss of other heat-labile nutrients, thereby lowering nutritional value [19]. Thermal treatments can also alter the color and functional properties of flour, including water-holding capacity and oil-holding capacity, which are important parameters for the development of food products [20,21]. Although recent studies have examined the effects of thermal processing on jackfruit seed powder, protein concentrate, and beverage products, these works have mainly focused on seed kernels or products prepared after removing the brown seed coat [22,23,24]. Therefore, the contribution of brown seed coat retention, particularly in combination with common thermal treatments such as boiling and steaming, to the nutritional, bioactive, antioxidant, and functional properties of jackfruit seed flour remains insufficiently understood.

The growing demand for natural, plant-derived ingredients with functional properties further highlights the importance of investigating jackfruit seed flour as a potential alternative to conventional cereal-based flours, particularly given its naturally gluten-free nature. Understanding the combined effects of seed coat retention and commonly applied thermal treatments, particularly boiling and steaming, is essential for optimizing the nutritional and functional quality of jackfruit seed flour. Therefore, the present study aimed to investigate the effects of brown seed coat retention and thermal processing on the color attributes, nutritional composition, β-glucan content, bioactive compound profiles, antioxidant activity, and functional properties of jackfruit seed flour produced from raw, boiled, and steamed seeds. This study provides new insights into the value of retaining the brown seed coat during flour production and supports the sustainable utilization of jackfruit seed, an underutilized fruit-processing by-product, for further development as a functional food ingredient.

## 2. Materials and Methods

### 2.1. Preparation of Jackfruit Seed Flour

**Figure 1:**
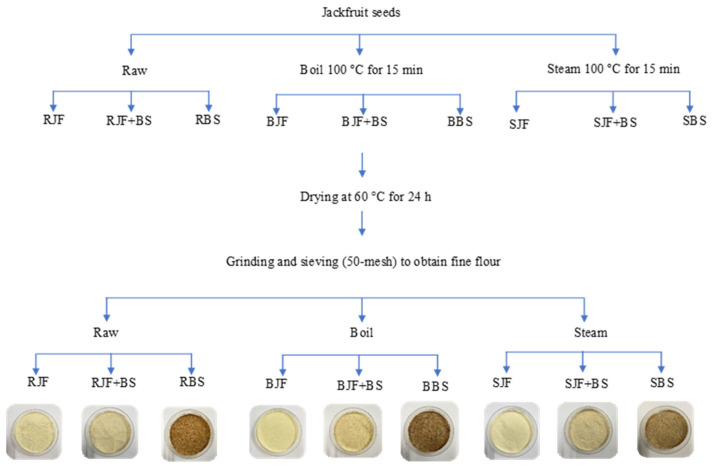
Preparation of jackfruit seed flour samples. R = raw; B = boiled; S = steamed; JF = jackfruit seed flour without the brown seed coat; BS = brown seed coat. (RJF = raw jackfruit seed flour; RJF + BS = raw jackfruit seed flour with the brown seed coat retained; RBS = raw brown seed coat; BJF = boiled jackfruit seed flour; BJF + BS = boiled jackfruit seed flour with the brown seed coat retained; BBS = boiled brown seed coat; SJF = steamed jackfruit seed flour; SJF + BS = steamed jackfruit seed flour with the brown seed coat retained; SBS = steamed brown seed coat).

Jackfruit seeds (*Artocarpus heterophyllus* Lam.) were prepared using a modified method based on previous studies [7,13]. Briefly, damaged and germinated seeds were removed prior to processing. Jackfruit seeds were obtained from a single seed lot to minimize lot-to-lot variation and were divided according to the processing treatments, including raw, boiled, and steamed samples. The seeds were then subjected to boiling or steaming at 100 °C for 15 min, while raw jackfruit seeds were used as the control group. Following each thermal treatment, the seeds were separated into three groups: seeds with the brown seed coat removed, seeds with the brown seed coat retained, and isolated brown seed coat samples (Figure 1). All samples were washed with distilled water and cut into approximately 1 cm pieces. The samples were subsequently dried in a hot air oven at 60 °C for 24 h, ground, and passed through a 50-mesh sieve to obtain fine flour, which was stored for further analysis. Commercial rice flour (Thai Flour Industry Co., Ltd., Bangkok, Thailand) and wheat flour (United Flour Mill Public Co., Ltd., Samut Prakan, Thailand) were also included as reference samples for comparison. However, since these flours differ from jackfruit seed flour in botanical origin, degree of refinement, composition, and processing conditions, this comparison should be interpreted only as a reference point rather than direct technological equivalence. The abbreviations used throughout this study are as follows: RJF = raw jackfruit seed flour; RJF + BS = raw jackfruit seed flour with the brown seed coat retained; RBS = raw brown seed coat; BJF = boiled jackfruit seed flour; BJF + BS = boiled jackfruit seed flour with the brown seed coat retained; BBS = boiled brown seed coat; SJF = steamed jackfruit seed flour; SJF + BS = steamed jackfruit seed flour with the brown seed coat retained; SBS = steamed brown seed coat; RF = rice flour; WF = wheat flour.

### 2.2. Instrumental Evaluation of the Color of Jackfruit Seeds Flour

Instrumental color analysis of the flour samples was performed using a CR-400 Chroma Meter (Konica Minolta Inc., Osaka, Japan) according to the manufacturer’s instructions. Color parameters were recorded in the CIELAB color system, where L* corresponds to lightness, a* represents the greenish–reddish color coordinate, and b* represents the bluish–yellowish color coordinate [25].

### 2.3. Determination of Water Activity (a_w_)

The water activity (a_w_) was measured using an AquaLab meter (Decagon Devices, Pullman, WA, USA) according to the manufacturer’s instructions.

### 2.4. Proximate Analysis

Moisture (925.10), ash (900.02), lipid (320.176), protein (920.177), and fiber (985.29) contents were determined according to AOAC methods as described by Boonarsa et al. (2025) [26]. Protein content was analyzed using the Kjeldahl method, while lipid content was determined by Soxhlet extraction with petroleum ether as the solvent. Fiber content was measured using the gravimetric method. Total carbohydrate content was calculated by difference after subtracting the contents of moisture, ash, lipid, protein, and fiber from 100. All results are expressed as percentages on a wet-weight basis.

### 2.5. Evaluation of Antioxidant Properties

#### 2.5.1. Extraction Procedure

Approximately 1 g of samples was homogenized with 30 mL of 99.99% methanol. The mixture was agitated at 150 rpm for 12 h at room temperature. The solution was then filtered through Whatman No. 1 filter paper to obtain the extract for further analysis, following a modified method from a previous study [27].

#### 2.5.2. Determination of Total Phenolic Content (TPC)

The total phenolic content was quantified using the Folin–Ciocalteu colorimetric method, following a previously reported protocol [28]. In brief, 200 μL of the extract was mixed with 300 μL of 10% Folin–Ciocalteu reagent and incubated for 5 min. Then, 2.25 mL of 6% sodium carbonate (Na_2_CO_3_) solution was added. The reaction mixture was left at ambient temperature for 90 min, and absorbance was measured at 725 nm using a UV–visible spectrophotometer (DR 2700, HACH, Loveland, CO, USA). Results are expressed as mg of gallic acid equivalents per g of sample (mg GAE/g).

#### 2.5.3. Determination of Total Flavonoid Content (TFC)

The total flavonoid content was assessed according to a published method [27]. A reaction mixture containing 500 μL of extract, 2.25 mL of distilled water, and 150 μL of 5% sodium nitrite (NaNO_2_) was incubated for 6 min. Subsequently, 300 μL of 10% aluminum chloride (AlCl_3_) was added and allowed to stand for 5 min. Finally, 100 μL of 1 mol/L sodium hydroxide (NaOH) was introduced. The absorbance was read at 510 nm, and results are reported as mg of quercetin equivalents per g of sample (mg QE/g).

#### 2.5.4. DPPH Free Radical Scavenging Activity

The antioxidant capacity of the extracts was evaluated using the DPPH (2,2-diphenyl-1-picrylhydrazyl) assay, following a previously published method [28]. A 500 μL aliquot of each extract was mixed with 4.5 mL of a 0.1 mmol/L DPPH solution in methanol. The mixture was incubated in the dark for 30 min, and the absorbance was read at 517 nm. The scavenging activity is expressed as mg vitamin C equivalents per g of sample (mg VCE/g).

#### 2.5.5. Ferric Reducing Antioxidant Power (FRAP) Assay

Antioxidant activity was measured using the ferric reducing antioxidant power (FRAP) assay, following the method described by Taesuk et al. (2025) [29], with slight modifications. Briefly, 0.1 mL of the extracted sample was mixed with 4.5 mL of FRAP reagent. The mixture was incubated in the dark at ambient temperature for 10 min, after which the absorbance was measured at 593 nm. The results are expressed as mg of ferrous sulfate equivalents per g of sample (mg FeSO_4_ equivalents/g).

### 2.6. Determination of β-Glucan

β-Glucan content was determined using the Megazyme β-Glucan Assay Kit (K-BGLU, Megazyme, Bray, Ireland) according to the manufacturer’s instructions. This assay is specific for mixed-linkage [(1→3)(1→4)]-β-D-glucan. Briefly, samples were suspended and hydrated in buffer solution at pH 6.5, followed by incubation with purified lichenase. The mixture was then hydrolyzed to completion using purified β-glucosidase. The released D-glucose was quantified colorimetrically using a glucose oxidase/peroxidase reagent. All measurements were conducted in triplicate.

### 2.7. Determination of Phenolic Content and Flavonoid Content

Phenolic acid and flavonoid profiling was performed using the HPLC system (Shimadzu, Kyoto, Japan) following previously published methods [30,31]. The system was equipped with a guard column and an InertSustain^®^ C18 column (250 mm × 4.6 mm i.d., 5 µm; GL Sciences Inc., Tokyo, Japan). The detection wavelengths were 280 nm, 320 nm, and 370 nm for hydroxybenzoic acid, hydroxycinnamic acids, and flavonoids, respectively. Compounds in the extracts were identified and quantified using external standards of phenolic acids and flavonoids. The validation parameters, including calibration ranges, regression equations, R^2^, LOD, LOQ, and recovery, are provided in Supplementary Table S1. Heatmap visualization of individual phenolic acid and flavonoid profiles was performed using the “heatmaply” package [32] in R version 4.5.1 to compare compound abundance patterns across samples. Data were screened prior to analysis, and values were excluded only when chromatographic interference, including overlapping peaks and excessive baseline noise, prevented reliable peak integration and accurate quantification.

### 2.8. Analysis of Functional Properties

#### 2.8.1. Water Solubility Index (WSI)

The water solubility index (WSI) was assessed following a published procedure [33]. Specifically, 2.5 g of each sample was mixed with 25 g of distilled water and stirred for 30 min. The resulting dispersion was transferred into pre-weighed 50 mL centrifuge tubes, adjusted to a final weight of 32.5 g, and centrifuged at 3000× *g* for 10 min. After centrifugation, the sediment was weighed, while the supernatant was collected to determine the amount of dissolved solids. The WSI was then calculated using Equation (1).

(1) $$\mathrm{WSI}\;(\%)=\frac{Weight\;of\;dissolved\;solids\;in\;supernatant}{Weight\;of\;dry\;solids}\times 100$$

#### 2.8.2. Water-Holding Capacity (WHC) and Water Swelling Capacity (WSC)

The water-holding capacity (WHC) was measured following the method described by He et al. (2023) [34], while the water swelling capacity (WSC) was assessed with slight modifications to suit the properties of jackfruit seed flour samples. Approximately 0.1 g of dried sample was placed into a 15 mL centrifuge tube, followed by the addition of 10 mL of deionized water. The mixture was left to stand at 25 °C for 24 h. The initial volume of the dry sample (V_0_) and the volume after hydration (V_1_) were recorded. The sample was then centrifuged at 3000× *g* for 20 min to remove the supernatant, and the mass of the tube along with the remaining precipitate (m_2_) was measured. WHC was calculated using the following formula:

(2) $$\mathrm{WHC}\;(g/g)=\frac{m_{2}-m_{1}}{m_{0}}\;$$

where m_0_ refers to the mass of the dried sample; m_1_ refers to the mass of the centrifuge tube; and m_2_ refers to the mass of the centrifuge tube and precipitate.

Water swelling capacity (WSC): WSC was calculated using the following:

(3) $$\mathrm{WSC}\;(\mathrm{mL}/g)=\frac{v_{1}-v_{0}}{m_{0}}\;$$

where v_0_ refers to the volume of the dried sample; v_1_ refers to the volume of the hydrated sample; and m_0_ refers to the mass of the dried sample.

#### 2.8.3. Oil-Holding Capacity (OHC)

The OHC was measured based on the method described by He et al. (2023) [34], with slight modifications for jackfruit seed flour samples. Approximately 0.2 g of the dried sample was placed into a 15 mL centrifuge tube, followed by the addition of 5 mL of soybean oil. The mixture was thoroughly blended and allowed to stand at 25 °C for 24 h. It was then centrifuged at 3500× *g* for 30 min. Excess oil adhering to the tube walls was carefully removed using oil-absorbent paper, and the combined mass of the precipitate and tube was recorded. The OHC was then calculated using the following equation:

(4) $$\mathrm{OHC}\;(g/g)=\frac{m_{2}-m_{1}}{m_{0}}\;$$

where m_0_ refers to the mass of the dried sample; m_1_ refers to the centrifuge tube; and m_2_ refers to the mass of the centrifuge tube and the observed oil samples.

### 2.9. Statistical Analysis

All results were analyzed in triplicate using separate aliquots or subsamples from the corresponding prepared extract or flour sample, depending on the assay. Results are expressed as mean ± standard deviation (SD). Statistical analysis was performed using IBM SPSS Statistics version 17.0. Since the study included isolated brown seed coat samples and reference flours that did not fit into a complete two-factor factorial design, one-way analysis of variance (ANOVA) followed by Duncan’s multiple-range test (DMRT) was used to compare all sample groups. Interpretations related to processing method and brown seed coat retention were based on direct comparisons among the corresponding sample groups. Differences were considered statistically significant at *p* < 0.05.

## 3. Results and Discussion

### 3.1. Water Activity (a_w_) and Color Characteristics

The water activity (a_w_) of jackfruit seed flour varied considerably depending on the processing methods and brown seed coat retention, with values ranging from 0.25 to 0.44 (Table 1). Raw jackfruit seed flour (RJF) exhibited a moderate a_w_ of 0.37, whereas RJF + BS, in which the brown seed coat was retained, showed a higher value of 0.44. In contrast, boiled jackfruit seed flour (BJF) and steamed jackfruit seed flour (SJF) samples recorded lower a_w_ values (0.30–0.32), likely reflecting starch gelatinization and redistribution of water during heating [35]. Among all samples, rice flour (RF) and wheat flour (WF) presented the highest a_w_ (0.53–0.57). While reduced a_w_ enhances shelf-life stability by restricting microbial growth, extremely low a_w_ may also hinder powder dispersibility and hydration in flour blends [36].

Color parameters also demonstrated clear differences. Jackfruit seed flour samples without the brown seed coat (RJF, BJF, and SJF) showed higher lightness values (L* = 87.25–88.43) than those with the brown seed coat retained (L* = 80.09–83.21), indicating that retention of the brown seed coat contributed to reduced flour brightness through its pigmentation. In comparison, RF (L* = 95.94) and WF (L* = 93.42) exhibited the highest lightness values among all samples, possibly due to flour refinement and the absence of pigmented outer layers. In terms of redness (a*), jackfruit seed flour with the brown seed coat retained exhibited significantly higher red color values (a* = 2.28–2.90) than seed coat-free flour samples, which remained nearly neutral (a* = 0.04–0.77). The raw brown seed coat (RBS) showed the highest redness value (a* = 7.60), possibly due to the presence of pigments, polyphenols, and tannins in the brown seed coat [37,38], which contribute to darker coloration. Regarding yellowness (b*), flour seed coat-free flour samples generally exhibited higher values (b* = 14.42–15.43) than those with the brown seed coat retained (b* = 12.98–14.91), possibly reflecting the natural yellowish color associated with starch-rich flour components. Overall, brown seed coat retention influenced the color characteristics of jackfruit seed flour by reducing lightness and increasing redness. These findings suggest that the brown seed coat plays an important role in determining the visual appearance and color attributes of jackfruit seed flour. The resulting color changes may influence consumer perception and product acceptability, depending on the intended application. A darker, more reddish color may be acceptable or even desirable in products such as whole-grain bakery products, cookies, or cocoa-flavored formulations, whereas lighter-colored products may require lower inclusion levels or further color adjustment.

### 3.2. Proximate Composition and β-Glucan Content

The proximate composition of jackfruit seed flour samples is presented in Table 2. Significant variations (*p* < 0.05) were observed among treatments in moisture, ash, protein, fat, fiber, and carbohydrate contents. Moisture content ranged from 1.23% in raw brown seed coat (RBS) to 13.31% in steamed jackfruit seed flour (SJF). The higher moisture content observed in boiled and steamed samples compared to raw samples may be attributed to water absorption during thermal processing and structural changes associated with starch gelatinization. During gelatinization, disruption of the crystalline starch structure and hydrogen bonds facilitates water diffusion into the amorphous regions of starch granules, promoting granule swelling and increased water absorption [39,40]. These processes may subsequently influence the moisture content of the final flour products.

Ash content was highest in raw brown seed coat (RBS, 6.50%) and boiled brown seed coat (BBS, 5.99%), indicating that the seed coat fraction is richer in minerals. Overall, jackfruit seed flour samples generally showed higher ash contents than the reference flours, rice flour (RF, 0.26%) and wheat flour (WF, 0.79%), supporting previous reports that jackfruit seeds serve as a valuable source of minerals compared with conventional cereal flours [41]. Protein content also varied significantly, ranging from 12.57% to 18.18%. Overall, jackfruit seed flour samples exhibited higher protein contents than rice flour (7.65%) and wheat flour (11.46%). Samples with the brown seed coat retained generally exhibited higher protein levels, suggesting that the brown seed coat may contribute additional protein components to the flour. Fat content was relatively low in most jackfruit seed flour samples (1.00–2.94%) and was lower than that of wheat flour (5.79%). Crude fiber content ranged from 2.58% to 8.39%. The brown seed coat exhibited higher crude fiber content; therefore, samples with the brown seed coat retained consistently showed higher crude fiber contents than seed coat-free samples, indicating that the brown seed coat is a major source of fiber. Similar findings have been reported in previous studies, which showed that seed coats are rich sources of fiber [42,43]. Carbohydrates represented the predominant macronutrient in jackfruit seed flour samples, ranging from 62.36% to 75.69%, which was comparable to rice flour (78.99%) and wheat flour (69.67%).

The β-glucan content of the jackfruit seed flour samples ranged from 56.11 to 286.71 mg/g, with the highest concentration observed in raw jackfruit seed flour with the brown seed coat retained (RJF + BS), suggesting that the seed coat retention may enhance the level of bioactive polysaccharides. Conversely, boiled and steamed samples exhibited markedly lower β-glucan levels, likely due to leaching of soluble solids into the processing water during thermal treatment. Thermal processing may disrupt the food matrix and promote the release of water-soluble components [44], including β-glucan, potentially leading to reductions in the measured β-glucan content. Interestingly, steamed samples generally exhibited higher β-glucan contents than boiled samples. This may be attributed to the greater leaching of soluble components during boiling compared with steaming [44]. As β-glucan is a soluble fiber, it may be more susceptible to loss into the cooking water during boiling than during steaming. Further analysis of cooking water composition and solid losses would provide additional evidence to support this proposed mechanism. It should also be noted that the values reported in this study represent mixed-linkage [(1→3)(1→4)]-β-D-glucan determined using the Megazyme K-BGLU enzymatic assay, which is commonly applied to cereal-based matrices but has not been specifically validated for jackfruit seed flour. Therefore, possible matrix-related effects should be considered when interpreting the β-glucan values.

Overall, both processing conditions and seed coat retention significantly affected the nutritional composition of jackfruit seed flour samples. In general, jackfruit seed flour possesses promising nutritional characteristics compared with conventional cereal flours, including higher ash, protein, and fiber contents. Moreover, retention of the brown seed coat further enhanced the fiber level, supporting its potential application as a value-added functional ingredient.

### 3.3. Total Phenolic Content, Total Flavonoid Content, and Antioxidant Activities

**Figure 2:**
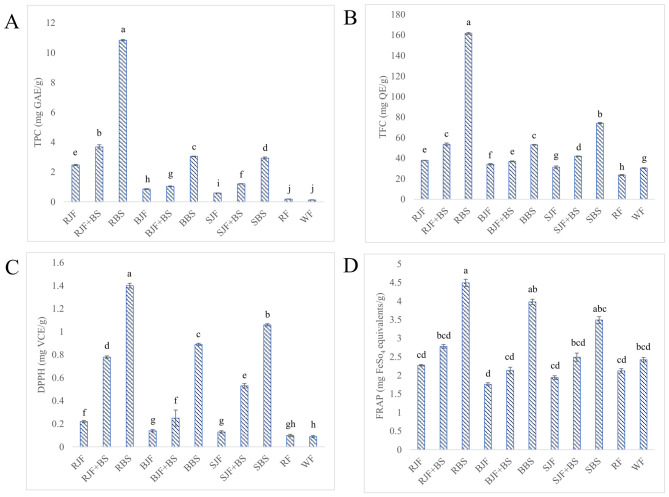
Determination of (**A**) total phenolic content, (**B**) total flavonoid content, (**C**) DPPH antioxidant activity, and (**D**) FRAP antioxidant activity. Different letters (a−j) indicate significant differences (*p* < 0.05) between samples.

The phenolic and flavonoid contents, as well as antioxidant activities, of jackfruit seed flour samples varied significantly depending on processing method and brown seed coat retention (Figure 2 and Supplementary Table S2). Overall, brown seed coat samples (RBS, BBS, and SBS) exhibited the highest TPC, TFC, and DPPH antioxidant activities, followed by jackfruit seed flour samples with the brown seed coat retained (RJF + BS, BJF + BS, and SJF + BS), whereas seed coat-free flour samples (RJF, BJF, and SJF) showed the lowest values. A similar trend was observed for FRAP activity, although the differences were less pronounced. Raw samples generally exhibited higher bioactive compound contents and antioxidant activities than thermally processed samples. Raw jackfruit seed flour (RJF) exhibited TPC and TFC values of 2.47 mg GAE/g and 37.92 mg QE/g, respectively, while retention of the brown seed coat in RJF + BS significantly increased these values to 3.69 mg GAE/g and 53.68 mg QE/g. Among all samples, raw brown seed coat (RBS) showed the highest TPC (10.84 mg GAE/g), TFC (161.67 mg QE/g), and antioxidant activities measured by DPPH (1.40 mg VCE/g) and FRAP (4.49 mg FeSO_4_ equivalents/g), suggesting that the brown seed coat is a major contributor to the antioxidant potential of jackfruit seed flour.

Thermal processing, either boiling or steaming, reduced bioactive compound contents and antioxidant activities across most jackfruit seed flour samples, likely due to degradation and possible leaching of bioactive compounds during heat treatment [45,46,47]. These findings are consistent with previous studies demonstrating that thermal processing can reduce bioactive compounds and antioxidant activity [47,48]. However, future studies examining the composition of cooking water and solid losses could further clarify the contribution of leaching to these changes. Among the thermally treated samples, boiled and steamed brown seed coat (BBS and SBS, respectively) exhibited the highest TPC, TFC, and antioxidant activities, followed by jackfruit seed flour with the brown seed coat retained (BJF + BS and SJF + BS), whereas seed coat-free jackfruit seed flour samples (BJF and SJF) showed the lowest values.

Compared with reference flours (RF and WF), jackfruit seed flour showed greater bioactive potential, particularly in brown seed coat samples and flour samples with the brown seed coat retained. These findings are consistent with previous studies showing that different seed or grain fractions display different bioactive properties, with the outer layers generally exhibiting higher antioxidant potential than the inner endosperm [49,50,51]. Overall, the brown seed coat served as an important contributor to the bioactive compound content and antioxidant activity of jackfruit seed flour. Although thermal processing reduced bioactive and antioxidant levels, retaining the brown seed coat during flour production resulted in higher levels of bioactive compounds, supporting its potential application as a natural antioxidant ingredient in functional and health-oriented food products. However, antinutritional compounds such as tannins, phytates, and enzyme inhibitors should be further investigated before confirming its practical application in food products.

### 3.4. Phenolic Acid and Flavonoid Composition

**Figure 3:**
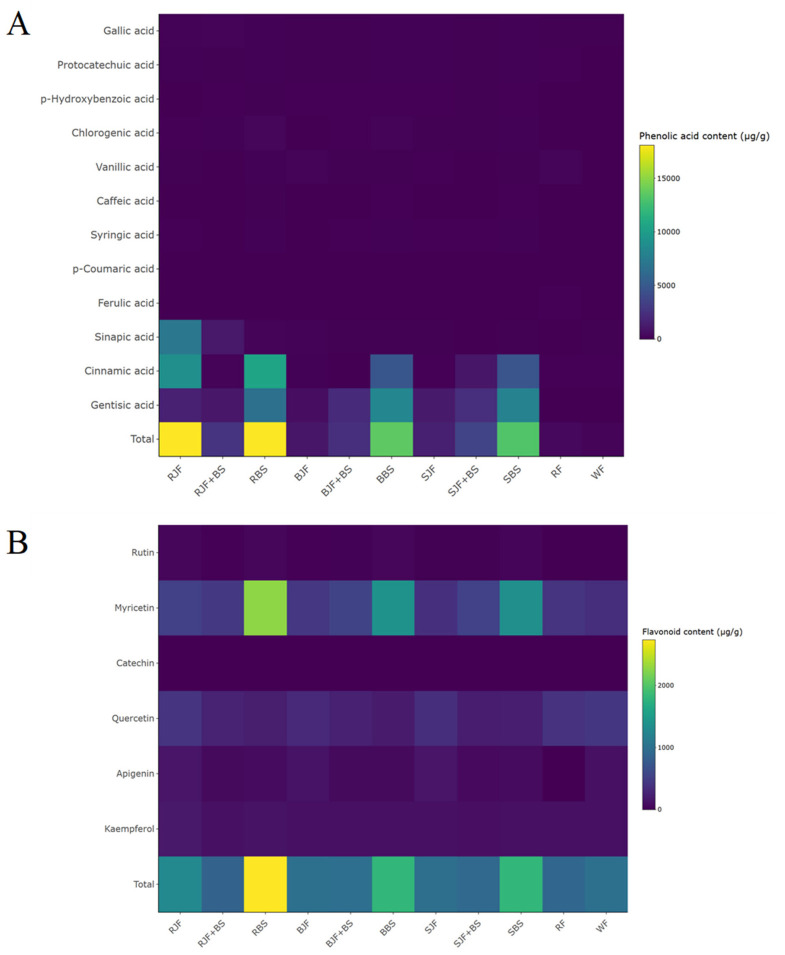
Heatmap analysis illustrating the distribution patterns of (**A**) individual phenolic acids and (**B**) flavonoids across jackfruit seed flour samples.

Phenolic compounds, including flavonoids, play a central role in free radical scavenging and reducing activity. These compounds exert their health-promoting effects through multiple mechanisms, primarily as potent antioxidants and enzyme inhibitors [6,52]. Flavonoids, one of the most abundant groups of plant secondary metabolites, are widely distributed throughout the plant kingdom, particularly in fruits and vegetables. Their consumption has been strongly associated with numerous health benefits and protection against various chronic diseases [53]. In the present study, heatmap analysis illustrates the distribution patterns of individual phenolic acids and flavonoids across jackfruit seed flour samples prepared under different processing conditions and with or without the brown seed coat retention (Figure 3A).

Distinct differences were observed between seed coat-free samples and samples with the brown seed coat retained, as well as among raw, boiled, and steamed samples, indicating that both thermal processing and brown seed coat retention influenced the phenolic composition of jackfruit seed flour (Figure 3A and Supplementary Table S3). Among the brown seed coat samples, raw brown seed coat (RBS) exhibited the highest total identified phenolic acid content, whereas boiled brown seed coat (BBS) and steamed brown seed coat (SBS) showed lower levels. This trend was generally consistent with the TPC results shown in Figure 2. Cinnamic acid and gentisic acid were the predominant phenolic acids detected in the brown seed coat samples, suggesting that these compounds may contribute substantially to the phenolic profile of the seed coat. For thermally processed samples, boiled (BJF + BS) and steamed (SJF + BS) jackfruit seed flour samples with the brown seed coat retained showed higher total identified phenolic acid contents than their corresponding seed coat-free samples (BJF and SJF). This observation was consistent with the TPC results (Figure 2), suggesting that the brown seed coat contributed to the higher phenolic acid levels observed in thermally processed samples.

In contrast, a different pattern was observed in raw samples. RJF showed the highest total identified phenolic acid content, while RBS exhibited a slightly lower but comparable level. Unexpectedly, RJF + BS showed a markedly lower total identified phenolic acid content than RJF, although retention of the brown seed coat was expected to increase phenolic acid levels. This result contrasted with the TPC data in Figure 2 and with the trends observed in boiled and steamed samples. This discrepancy may be explained by methodological differences between colorimetric TPC analysis and HPLC-based quantification, as TPC reflects the overall reducing capacity of extractable compounds, including phenolics [54,55], whereas HPLC quantifies only selected individual phenolic acids. In addition, interactions between phenolic compounds and flour matrix components, including starch, protein, and fiber [56,57,58], may influence the extractability and recovery of individual phenolic acids in RJF + BS. Nevertheless, all jackfruit seed flour samples contained higher levels of total identified phenolic acids than the reference flours, rice flour (RF) and wheat flour (WF), which was consistent with their lower TPC values shown in Figure 2.

Flavonoids also showed distinct distribution patterns among the samples (Figure 3B and Supplementary Table S3). The highest total identified flavonoid contents were observed in the brown seed coat samples (RBS, BBS, and SBS), which is generally consistent with the TFC results shown in Figure 2. Myricetin and quercetin were the predominant flavonoids detected in jackfruit seed flour samples, with myricetin being more abundant in the brown seed coat samples (RBS, BBS, and SBS), whereas quercetin was more prominent in seed coat-free jackfruit seed flour samples (RJF, BJF, and SJF). Interestingly, the trend observed for flavonoids differed from the expected pattern. Although retaining the brown seed coat was expected to increase flavonoid content, seed coat-free jackfruit seed flour samples (RJF, BJF, and SJF) showed higher total identified flavonoid contents than their corresponding samples with the brown seed coat retained (RJF + BS, BJF + BS, and SJF + BS). This pattern was observed not only in raw samples but also after boiling and steaming. A similar discrepancy was observed for phenolic acids in raw samples, where RJF + BS showed lower total HPLC-detected phenolic acid content than RJF despite retaining the brown seed coat. This flavonoid pattern may therefore be related to interactions between bioactive compounds and flour matrix components, including starch, protein, and fiber [56,57,58], which could influence the extractability and recovery of individual flavonoid compounds during HPLC analysis.

Thermal processing also influenced flavonoid content. Boiled and steamed samples generally showed lower levels of identified flavonoids in both brown seed coat samples and seed coat-free jackfruit seed flour samples, which may be attributed to thermal degradation and leaching of bioactive compounds during heat treatment [45,46,47]. However, jackfruit seed flour samples with the brown seed coat retained showed an opposite trend, as BJF + BS and SJF + BS exhibited higher total identified flavonoid contents than RJF + BS. This suggests that although flavonoids in jackfruit seed flour with the brown seed coat retained may be less extractable due to interactions between bioactive compounds and flour matrix components, thermal processing may partially disrupt these structures and improve the release or recovery of certain flavonoid compounds.

### 3.5. Functional Properties

Functional properties were evaluated for jackfruit seed flour samples without the brown seed coat (RJF, BJF, and SJF) and those with the brown seed coat retained (RJF + BS, BJF + BS, and SJF + BS), as presented in Table 3. The functional properties of jackfruit seed flour were strongly influenced by processing methods and brown seed coat retention. The water solubility index (WSI) was highest in raw jackfruit seed flour (RJF, 18.30%), whereas boiling and steaming significantly reduced WSI to 14.16–15.02%. This decrease may be attributed to the loss of soluble compounds during thermal processing, together with heat-induced protein denaturation, changes in molecular order, and new interactions among flour components, which may reduce the solubility of the remaining flour matrix [44,59,60,61].

Brown seed coat retention further reduced WSI in raw jackfruit seed flour (RJF + BS, 16.70%) compared with the seed coat-free sample (RJF), possibly due to the higher proportion of insoluble fiber and cell wall materials in the seed coat, which may limit the release of soluble solids. However, no significant differences were observed between boiled and steamed samples with and without the brown seed coat, suggesting that thermal processing may have modified the flour matrix through starch gelatinization, protein denaturation, and structural disruption [59,60,61], thereby reducing the relative influence of the brown seed coat retention on solubility. Steamed samples tended to show higher WSI than boiled samples, which may be related to lower leaching losses during steaming compared with boiling, allowing greater retention of soluble components in the final flour. By contrast, rice flour (RF) and wheat flour (WF) showed markedly lower WSI (6.52–6.81%), suggesting that jackfruit seed flour samples contain a greater proportion of soluble components, which may enhance its dispersibility in composite flour systems. Similar WSI values for jackfruit seed flour have been reported by Juárez-Barrientos et al. [62], which is consistent with the present results. From a technological perspective, the relatively high WSI of jackfruit seed flour suggests a greater proportion of water-soluble components, which may facilitate dispersion and reconstitution in composite flour systems, instant beverage powders, soup mixes, batters, and sauce formulations. However, product-specific studies are required to confirm its performance in these food systems.

Water-holding capacity (WHC) ranged from 3.66 to 6.31 g/g and was significantly affected by processing treatment and brown seed coat retention. The highest WHC was observed in boiled jackfruit seed flour with the brown seed coat retained (BJF + BS), whereas steamed jackfruit seed flour without the seed coat (SJF) exhibited the lowest value. In general, seed coat retention increased WHC, reflecting the contribution of fiber and structural polysaccharides that provide abundant hydrophilic functional groups capable of binding and retaining water through hydrogen bonding [63]. The WHC values obtained in the present study are consistent with those previously reported for jackfruit seed flour and other starch-rich plant flours, which generally range from approximately 2.0 to 6.0 g/g depending on cultivar, processing conditions, and analytical methodology [13,64]. WHC is an important functional property in food formulation because it influences the texture of the final product and reflects the ability of flour components to absorb water, which contributes to thickening and viscosity properties [65,66]. Compared with rice flour (2.95 g/g) and wheat flour (3.14 g/g), jackfruit seed flour generally demonstrated a higher water-holding capacity. This property may be advantageous in bakery products, composite flours, and gluten-free formulations, where water retention can contribute to hydration behavior, texture development, and product stability. However, product-specific studies are needed to confirm its effects on dough handling, final texture, yield, and storage quality.

Oil-holding capacity (OHC) ranged from 2.28 to 4.91 g/g. Raw jackfruit seed flour with the retained brown seed coat (RJF + BS) exhibited the highest OHC, whereas boiled jackfruit seed flour without the seed coat (BJF) showed the lowest value. Seed coat retention generally increased OHC in jackfruit seed flour samples. These OHC values are comparable with those reported for jackfruit seed flour and other protein- and fiber-rich plant flours [13,64]. Oil retention is influenced by hydrophobic interactions between lipids and nonpolar amino acid residues of proteins, together with the physical entrapment of oil within porous fiber structures. Consequently, flours with relatively high OHC may be advantageous in food applications where oil binding, flavor retention, mouthfeel, and structural stability are desirable. However, product-specific formulation studies are needed to confirm these functional benefits in different food matrices.

Water swelling capacity (WSC) did not differ significantly among jackfruit seed flour samples, with values ranging from 3.43 to 4.64 mL/g. Among these samples, steamed jackfruit seed flour (SJF) showed the highest value (4.64 mL/g), whereas wheat flour showed the lowest WSC value (3.25 mL/g). Unlike WSI, which reflects the release of soluble compounds into water, WSC is more closely associated with the ability of starch and insoluble polysaccharides to absorb and retain water. Therefore, although thermal processing reduced flour solubility, it had only a limited influence on the swelling capacity of the remaining starch-rich matrix. Similar relationships between WSI and WSC have been reported for starch-based flours, where swelling behavior depends predominantly on starch granule integrity and composition rather than on the concentration of soluble solids [8,13,67].

Overall, boiling and steaming generally reduced WSI compared with raw jackfruit seed flour, whereas the effect of brown seed coat retention on WSI was observed mainly in raw flour and became less apparent after thermal processing. Seed coat retention generally improved WHC and OHC, indicating the contribution of fiber- and polysaccharide-rich seed coat materials to water and oil retention. In contrast, WSC remained relatively stable among jackfruit seed flour samples, suggesting that these functional properties were not substantially affected by processing method or brown seed coat retention. Compared with rice and wheat flours, jackfruit seed flour exhibited higher WSI and WHC, with generally comparable OHC and WSC, indicating its potential as a functional ingredient in composite flour systems and other food formulations where hydration, dispersion, and water retention properties are desirable. However, product-specific studies are needed to confirm its technological performance in actual food systems.

## 4. Conclusions

This study demonstrated that brown seed coat retention and thermal processing significantly influenced the color, nutritional composition, β-glucan content, bioactive profiles, antioxidant activity, and functional properties of jackfruit seed flour. Brown seed coat retention reduced flour lightness and increased redness while also improving fiber content and enhancing phenolic, flavonoid, and antioxidant properties. Brown seed coat samples showed the highest TPC, TFC, and DPPH antioxidant activities, followed by flour samples with the brown seed coat retained, whereas seed coat-free samples showed the lowest values. These results suggest that the brown seed coat is an important contributor to the bioactive and antioxidant potential of jackfruit seed flour. Although boiling and steaming reduced bioactive compound contents and antioxidant activity, flour samples with the brown seed coat retained still showed higher bioactive potential than seed coat-free samples and reference flours. Among the thermally processed brown seed coat-retained jackfruit seed flours, steamed flour appeared to better preserve bioactive compounds, antioxidant activity, and β-glucan than the corresponding boiled flour. Heatmap analysis further showed that thermal processing and brown seed coat retention altered the distribution of individual phenolic acids and flavonoids. Differences between colorimetric and HPLC-based results may reflect interactions between bioactive compounds and flour matrix components, which could influence the extractability and recovery of individual compounds. Jackfruit seed flour also showed favorable nutritional characteristics compared with rice and wheat flours, including higher ash, protein, and fiber contents. In terms of functional properties, jackfruit seed flour showed higher WSI and WHC than the reference rice and wheat flours, while OHC and WSC were generally comparable. These findings suggest that jackfruit seed flour, particularly when produced with the brown seed coat retained, shows potential for further development as a nutritious and bioactive functional ingredient for composite flour-based food applications. Further studies on product formulation, sensory acceptability, digestibility, storage stability, and analysis using multiple seed lots and independent processing batches are recommended to evaluate lot-to-lot and batch-to-batch variability and to support the broader application of jackfruit seed flour in food product development.

## Figures and Tables

**Table 1:** Water activity (a_w_) and color parameters (CIELAB) of jackfruit seed flour samples.

Samples	a_w_	L*	a*	b*
RJF	0.37 ± 0.03 ^c^	87.25 ± 0.02 ^d^	0.77 ± 0.01 ^g^	14.42 ± 0.35 ^b^
RJF + BS	0.44 ± 0.01 ^b^	83.21 ± 0.32 ^e^	2.62 ± 0.06 ^e^	14.91 ± 0.38 ^ab^
RBS	0.38 ± 0.01 ^c^	52.34 ± 0.10 ^j^	7.60 ± 0.02 ^a^	12.61 ± 0.04 ^e^
BJF	0.30 ± 0.01 ^de^	88.43 ± 0.31 ^c^	0.04 ± 0.01 ^h^	15.43 ± 0.29 ^a^
BJF + BS	0.32 ± 0.01 ^cd^	80.09 ± 0.11 ^g^	2.90 ± 0.04 ^d^	12.98 ± 0.56 ^de^
BBS	0.31 ± 0.01 ^de^	55.82 ± 0.02 ^i^	4.72 ± 0.15 ^b^	13.05 ± 0.56 ^de^
SJF	0.32 ± 0.01 ^cd^	88.11 ± 0.03 ^c^	0.16 ± 0.01 ^f^	15.23 ± 0.03 ^a^
SJF + BS	0.30 ± 0.01 ^de^	81.67 ± 0.09 ^f^	2.28 ± 0.03 ^f^	13.73 ± 0.18 ^c^
SBS	0.25 ± 0.01 ^e^	60.89 ± 0.26 ^h^	3.66 ± 0.06 ^c^	13.36 ± 0.26 ^cd^
RF	0.53 ± 0.02 ^a^	95.94 ± 0.40 ^a^	−0.21 ± 0.02 ^i^	4.28 ± 0.15 ^g^
WF	0.57 ± 0.01 ^a^	93.42 ± 0.40 ^b^	0.03 ± 0.01 ^h^	7.74 ± 0.15 ^f^

Results are expressed as mean ± SD. Mean values in the same column with different superscript letters are significantly different (*p* < 0.05). R = raw; B = boiled; S = steamed; JF = jackfruit seed flour without the brown seed coat; BS = brown seed coat. (RJF = raw jackfruit seed flour; RJF + BS = raw jackfruit seed flour with the brown seed coat retained; RBS = raw brown seed coat; BJF = boiled jackfruit seed flour; BJF + BS = boiled jackfruit seed flour with the brown seed coat retained; BBS = boiled brown seed coat; SJF = steamed jackfruit seed flour; SJF + BS = steamed jackfruit seed flour with the brown seed coat retained; SBS = steamed brown seed coat; RF = rice flour; WF = wheat flour).

**Table 2:** Proximate composition and β-glucans of jackfruit seed flour samples.

Samples	Moisture	Ash	Protein	Fat	Fiber	Carbohydrate	β-Glucans (mg/g)
RJF	4.73 ± 1.02 ^e^	3.50 ± 0.46 ^d^	13.05 ± 0.37 ^cd^	1.30 ± 0.51 ^cd^	3.67 ± 0.19 ^e^	73.76 ± 2.15 ^b^	223.25 ± 26.31 ^b^
RJF + BS	7.07 ± 0.71 ^d^	4.28 ± 0.58 ^c^	13.35 ± 0.65 ^c^	1.10 ± 0.16 ^cd^	5.25 ± 0.12 ^c^	68.95 ± 1.50 ^cd^	286.71 ± 38.64 ^a^
RBS	1.23 ± 0.71 ^f^	6.50 ± 0.23 ^a^	15.12 ± 0.62 ^b^	2.94 ± 1.10 ^b^	8.33 ± 0.32 ^a^	65.87 ± 2.07 ^de^	172.84 ± 14.4 ^c^
BJF	7.05 ± 0.75 ^d^	1.12 ± 0.06 ^e^	12.57 ± 0.27 ^cd^	1.00 ± 0.24 ^cd^	2.58 ± 0.15 ^f^	75.69 ± 0.40 ^ab^	83.77 ± 11.97 ^e^
BJF + BS	7.89 ± 0.74 ^cd^	5.05 ± 0.19 ^b^	13.52 ± 0.63 ^c^	1.37 ± 0.26 ^cd^	3.67 ± 0.22 ^e^	68.50 ± 0.93 ^cd^	56.11 ± 6.94 ^e^
BBS	4.84 ± 0.43 ^e^	5.99 ± 0.33 ^a^	18.18 ± 1.01 ^a^	1.71 ± 0.27 ^bcd^	6.18 ± 0.16 ^b^	64.73 ± 1.77 ^e^	131.13 ± 13.11 ^d^
SJF	13.31 ± 0.79 ^a^	4.21 ± 0.08 ^c^	12.95 ± 1.39 ^cd^	1.23 ± 0.48 ^cd^	4.58 ± 0.30 ^d^	63.72 ± 2.15 ^e^	165.69 ± 23.67 ^cd^
SJF + BS	9.25 ± 0.66 ^bc^	3.70 ± 0.55 ^cd^	15.26 ± 1.43 ^b^	1.13 ± 0.57 ^cd^	5.05 ± 0.20 ^c^	65.61 ± 2.85 ^de^	179.19 ± 19.1 ^c^
SBS	4.93 ± 1.51 ^e^	4.04 ± 0.48 ^cd^	17.91 ± 0.68 ^a^	2.36 ± 2.10 ^bc^	8.39 ± 0.10 ^a^	62.36 ± 2.41 ^e^	152.87 ± 9.10 ^cd^
RF	10.48 ± 2.09 ^b^	0.26 ± 0.03 ^f^	7.65 ± 0.67 ^e^	0.49 ± 0.05 ^c^	2.13 ± 0.18 ^g^	78.99 ± 2.02 ^a^	50.85 ± 12.71 ^e^
WF	10.42 ± 1.37 ^b^	0.79 ± 0.05 ^ef^	11.46 ± 1.35 ^d^	5.79 ± 0.48 ^a^	1.86 ± 0.07 ^g^	69.67 ± 2.29 ^c^	155.86 ± 23.98 ^cd^

Results are expressed as mean ± SD. Mean values in the same column with different superscript letters are significantly different (*p* < 0.05). R = raw; B = boiled; S = steamed; JF = jackfruit seed flour without the brown seed coat; BS = brown seed coat. (RJF = raw jackfruit seed flour; RJF + BS = raw jackfruit seed flour with the brown seed coat retained; RBS = raw brown seed coat; BJF = boiled jackfruit seed flour; BJF + BS = boiled jackfruit seed flour with the brown seed coat retained; BBS = boiled brown seed coat; SJF = steamed jackfruit seed flour; SJF + BS = steamed jackfruit seed flour with the brown seed coat retained; SBS = steamed brown seed coat; RF = rice flour; WF = wheat flour).

**Table 3:** Functional properties of jackfruit seed flour samples.

Samples	WSI (%)	WHC (g/g)	OHC (g/g)	WSC (mL/g)
RJF	18.30 ± 0.33 ^a^	4.64 ± 0.52 ^bc^	4.05 ± 0.79 ^ab^	3.43 ± 0.49 ^ab^
RJF + BS	16.70 ± 1.40 ^b^	5.21 ± 0.72 ^ab^	4.91 ± 0.43 ^a^	4.08 ± 0.63 ^ab^
BJF	14.16 ± 0.36 ^d^	5.09 ± 0.72 ^b^	2.28 ± 0.55 ^d^	4.20 ± 0.53 ^ab^
BJF + BS	14.14 ± 0.04 ^d^	6.31 ± 0.69 ^a^	3.43 ± 0.25 ^bc^	3.87 ± 1.00 ^ab^
SJF	15.02 ± 0.22 ^cd^	3.66 ± 0.81 ^cd^	2.82 ± 0.74 ^cd^	4.64 ± 0.75 ^a^
SJF + BS	16.16 ± 1.26 ^bc^	5.58 ± 0.70 ^ab^	4.36 ± 0.36 ^ab^	4.15 ± 0.50 ^ab^
RF	6.81 ± 0.82 ^e^	2.95 ± 0.31 ^d^	4.43 ± 0.29 ^ab^	3.50 ± 0.34 ^ab^
WF	6.52 ± 0.56 ^e^	3.14 ± 0.33 ^d^	4.03 ± 0.84 ^ab^	3.25 ± 0.68 ^b^

Results are expressed as mean ± SD. Mean values in the same column with different superscript letters are significantly different (*p* < 0.05). R = raw; B = boiled; S = steamed; JF = jackfruit seed flour without the brown seed coat; BS = brown seed coat. (RJF = raw jackfruit seed flour; RJF + BS = raw jackfruit seed flour with the brown seed coat retained; BJF = boiled jackfruit seed flour; BJF + BS = boiled jackfruit seed flour with the brown seed coat retained; SJF = steamed jackfruit seed flour; SJF + BS = steamed jackfruit seed flour with the brown seed coat retained; RF = rice flour; WF = wheat flour).

## Data Availability

The original contributions presented in this study are included in the article/Supplementary Materials. Further inquiries can be directed to the corresponding author.

## Supplementary Materials

The following supporting information can be downloaded at: https://www.mdpi.com/article/10.3390/foods15142529/s1, Table S1. Validation parameters of the HPLC-DAD method for the quantification of phenolic acids and flavonoids. Table S2. Total phenolic content, total flavonoid content, and antioxidant activities of jackfruit seed flour samples. Table S3. Contents of individual phenolic acids and flavonoids in jackfruit seed flour samples.
