# Supplementary material for: Effects of Brown Seed Coat Retention and Thermal Processing on Nutritional Composition, Bioactive Compounds, Antioxidant Activity, and Functional Properties of Jackfruit Seed Flour
Source: Foods. 2026 Jul 17;15(14):2529. doi: 10.3390/foods15142529 (PMC13409598; doi:10.3390/foods15142529)
Supplement: Supplementary file 1 [file foods-15-02529-s001.zip › foods-4412155-supplementary.pdf]

Effects of Brown Seed Coat Retention and Thermal Processing on Nutritional Composition, Bioactive Compounds, Antioxidant Activity, and Functional Properties of Jackfruit Seed Flour

Theeraphan Chumroenphat, Nonthiwat Taesuk, Nidthaya Seephua, Apichaya Bunyatratchata

Supplementary Table

Supplementary Table S1. Validation parameters of the HPLC-DAD method for the quantification of phenolic acids and flavonoids.

Parameter	Calibration range (µg/mL)	Regression equation	R^2	LOD (ppm)	LOQ (ppm)	Recovery (%)
Phenolic acid content (µg/g DW)						
Gallic acid	0.59 - 300	y = 32282x - 153589	0.9960	0.106	19.667	98-102
Protocatechuic acid	0.09 - 90.90	y = 15030x + 1551.2	0.9999	0.028	2.099	97-101
p-hydroxybenzoic acid	0.59 - 300	y = 78883x - 73853	0.9983	0.085	7.375	93-103
Chlorogenic acid	0.59 - 300	y = 38055x - 151574	0.9975	0.109	4.853	80-110
Vanillic acid	0.59 - 300	y = 21320x - 5203	0.9986	0.091	6.344	94-101
Caffeic acid	2.34 - 37.50	y = 2221x - 306.5	0.9927	0.18	9.629	93-102
Syringic acid	4.69 - 300	y = 774.35x - 4162.6	0.9993	0.05	13.516	95-103
p-coumaric acid	0.59 - 300	y = 81159x - 165836	0.9992	0.046	8.486	97-101
Ferulic acid	0.59 - 300	y = 62448x - 172995	0.9987	0.069	13.926	98-103
Sinapic acid	0.09 - 100.00	y = 115344x + 15300	0.9998	0.045	2.753	99-101
Cinnamic acid	0.09 - 100.00	y = 199642x + 447539	0.9998	0.025	1.437	98-102
Gentisic acid	9.38-300	y = 12532x + 49873	0.9990	0.043	2.531	98-102
Flavonoid content (µg/g DW)						
Rutin	0.09 - 100.00	8126.4x - 5359.1	0.9986	0.723	9.744	99-101
Myricetin	0.09 - 100.00	y = 51979x - 3689.3	0.9996	7.617	15.388	97-110
Catechin	3.90-250	y = 7.4703x - 30.341	0.9967	18.53	56.15	99-99.67
Quercetin	0.09 - 100.00	y = 87144x + 156218	0.9999	1.023	1.706	94-101
Apigenin	6.25-100	y = 23380x - 14228	0.9992	0.523	1.189	96-102
Kaempferol	0.09 - 100.00	y = 125899x + 140269	0.9996	7.854	24.453	98-105

Supplementary Table S2. Total phenolic content, total flavonoid content, and antioxidant activities of jackfruit seed flour samples.

Samples	TPC (mg GAE/g)	TFC (mg QE/g)	DPPH (mg vitamin C/g)	FRAP (mg $FeSo_4$/g)
RJF	$2.47±0.04^{e}$	$37.92±0.11^{e}$	$0.22±0.01^{f}$	$2.27±0.02^{cd}$
RJF+BS	$3.69±0.14^{b}$	$53.68±1.12^{c}$	$0.78±0.01^{d}$	$2.78±0.05^{bcd}$
RBS	$10.84±0.05^{a}$	$161.67±0.82^{a}$	$1.40±0.02^{a}$	$4.49±0.10^{a}$
BJF	$0.86±0.03^{h}$	$34.11±0.76^{f}$	$0.14±0.01^{g}$	$1.76±0.04^{d}$
BJF+BS	$1.04±0.03^{g}$	$36.95±0.45^{e}$	$0.25±0.07^{f}$	$2.13±0.09^{cd}$
BBS	$3.05±0.02^{c}$	$53.11±0.28^{c}$	$0.89±0.01^{c}$	$3.98±0.07^{ab}$
SJF	$0.59±0.01^{i}$	$31.31±1.20^{g}$	$0.13±0.01^{g}$	$1.94±0.05^{cd}$
SJF+BS	$1.21±0.01^{f}$	$41.97±0.31^{d}$	$0.53±0.02^{e}$	$2.49±0.11^{bcd}$
SBS	$2.94±0.07^{d}$	$74.17±0.68^{b}$	$1.06±0.01^{b}$	$3.49±0.09^{abc}$
RF	$0.19±0.01^{j}$	$23.59±0.51^{h}$	$0.10±0.01^{gh}$	$2.12±0.06^{cd}$
WF	$0.13±0.01^{j}$	$30.40±0.42^{g}$	$0.09±0.01^{h}$	$2.42±0.06^{bcd}$

Results are expressed as mean ± SD. Mean values in the same column with different superscript letters are significantly different ($p < 0.05$). R = raw; B = boiled; S = steamed; JF = jackfruit seed flour without the brown seed coat; BS = brown seed coat. (RJF = raw jackfruit seed flour; RJF+BS = raw jackfruit seed flour with the brown seed coat retained; RBS = raw brown seed coat; BJF = boiled jackfruit seed flour; BJF+BS = boiled jackfruit seed flour with the brown seed coat retained; BBS = boiled brown seed coat; SJF = steamed jackfruit seed flour; SJF+BS = steamed jackfruit seed flour with the brown seed coat retained; SBS = steamed brown seed coat; RF = rice flour; WF = wheat flour).

Supplementary Table S3. Contents of individual phenolic acids and flavonoids in jackfruit seed flour samples.

Parameter	Samples										
	RJF	RJF+BS	RBS	BJF	BJF+BS	BBS	SJF	SJF+BS	SBS	RF	WF
**Phenolic acid content (µg/g)**											
Gallic acid	97.50±12.48	166.51±17.91	63.47±0.87	47.26±0.78	51.83±0.28	72.95±1.53	54.26±1.78	49.04±1.38	65.32±0.32	48.62±0.13	47.36±0.38
Protocatechuic acid	77.78±6.90	39.90±1.69	71.29±4.14	36.77±0.45	48.98±0.67	60.74±0.77	80.26±3.16	36.14±0.13	65.18±1.03	35.56±1.15	0.79±0.09
p-Hydroxybenzoic acid	11.76±0.29	21.71±1.92	45.08±0.53	28.46±0.13	31.40±1.33	26.99±1.24	34.91±0.64	37.96±0.85	31.81±0.04	ND	ND
Chlorogenic acid	28.88±6.97	75.25±7.60	272.23±4.72	15.29±0.29	18.06±0.47	127.53±1.99	44.70±1.56	43.48±0.85	86.16±1.85	ND	ND
Vanillic acid	39.82±6.92	7.80±0.82	84.77±10.27	144.47±2.41	38.31±0.58	45.48±2.72	34.22±1.85	3.21±1.26	44.6±11.15	201.83±11.60	ND
Caffeic acid	5.26±0.19	2.30±0.12	48.69±0.53	0.68±0.02	2.10±0.06	20.63±0.30	0.67±0.02	0.65±0.02	20.62±0.10	ND	ND
Syringic acid	32.74±0.70	8.02±0.42	53.99±10.98	15.82±0.04	23.39±0.24	67.80±0.24	23.32±0.15	25.91±0.99	61.38±0.45	ND	ND
p-Coumaric acid	0.89±0.08	2.19±1.91	3.44±0.05	0.54±0.04	0.91±0.14	2.10±0.02	0.94±0.05	1.18±0.06	3.77±1.53	0.27±0.03	ND
Ferulic acid	10.79±0.91	1.95±0.26	1.21±0.20	4.14±0.45	14.96±1.97	2.48±0.10	5.50±0.42	6.55±0.27	9.81±0.12	20.02±4.48	17.19±0.16
Sinapic acid	7,145.77±1,074.61	1,137.86±347.04	172.50±2.24	93.74±77.57	45.35±9.66	48.44±10.49	37.11±5.07	5.24±0.01	39.3±1.97	12.45±5.91	51.95±0.96
Cinnamic acid	8,989.08±3,784.35	150.60±2.87	10,556.43±3,846.38	72.03±62.40	1.64±0.02	4,808.17±49.06	23.71±1.95	974.88±214.23	4,685.86±14.81	32.19±7.96	33.24±0.84
Gentisic acid	1,619.11±40.44	1,072.20±21.21	6,618.88±322.15	555.55±12.94	2,138.57±9.52	8,300.56±173.54	1,211.58±33.24	2,430.6±33.91	8,014.92±37.29	ND	ND
Total	18,059.38	2,686.29	17,991.98	1,014.75	2,415.5	13,583.87	1,551.18	3,614.84	13,128.73	350.94	150.53
**Flavonoid content (µg/g)**											
Rutin	44.17±13.83	3.20±1.12	43.32±7.00	4.89±1.74	9.06±1.04	38.09±2.66	5.54±0.21	6.46±0.45	33.88±0.28	ND	ND
Myricetin	517.90±32.12	435.34±7.80	2,270.67±86.13	430.78±60.41	542.31±17.94	1,389.91±103.21	376.23±33.09	534.52±18.59	1,365.67±884.29	409.6±36.94	353.97±5.71
Catechin	ND	ND	ND	ND	ND	ND	ND	ND	ND	ND	ND
Quercetin	407.78±27.14	249.97±13.11	217.69±44.01	312.31±50.90	246.21±6.89	196.93±24.88	356.34±34.08	216.41±20.50	220.77±37.05	389.87±41.06	424.03±62.73
Apigenin	146.81±37.64	64.54±3.63	74.22±3.43	132.48±12.92	67.00±2.03	66.30±1.26	146.82±9.69	60.31±1.35	69.72±4.22	ND	104.54±36.44
Kaempferol	172.02±33.20	106.97±6.97	127.17±1.18	111.68±7.46	108.11±1.15	108.27±1.41	104.75±8.78	94.01±2.15	105.64±2.23	105.48±2.41	109.66±11.28
Total	1288.68	860.02	2733.07	992.14	972.69	1799.50	989.68	911.71	1795.68	904.95	992.20

Results are expressed as mean ± SD; ND: Not detected. R = raw; B = boiled; S = steamed; JF = jackfruit seed flour without the brown seed coat; BS = brown seed coat. (RJF = raw jackfruit seed flour; RJF+BS = raw jackfruit seed flour with the brown seed coat retained; RBS = raw brown seed coat; BJF = boiled jackfruit seed flour; BJF+BS = boiled jackfruit seed flour with the brown seed coat retained ; BBS = boiled brown seed coat; SJF = steamed jackfruit seed flour; SJF+BS = steamed jackfruit seed flour with the brown seed coat retained; SBS = steamed brown seed coat; RF = rice flour; WF = wheat flour).
